# X-Ray Structure Reveals a New Class and Provides Insight into Evolution of Alkaline Phosphatases

**DOI:** 10.1371/journal.pone.0022767

**Published:** 2011-07-28

**Authors:** Subhash C. Bihani, Amit Das, Kayzad S. Nilgiriwala, Vishal Prashar, Michel Pirocchi, Shree Kumar Apte, Jean-Luc Ferrer, Madhusoodan V. Hosur

**Affiliations:** 1 Solid State Physics Division, Bhabha Atomic Research Centre, Trombay, Mumbai, India; 2 Molecular Biology Division, Bhabha Atomic Research Centre, Trombay, Mumbai, India; 3 Groupe Synchrotron, Institut de Biologie Structurale J-P Ebel, CEA-CNRS-UJF, Grenoble, France; University of South Florida, United States of America

## Abstract

The alkaline phosphatase (AP) is a bi-metalloenzyme of potential applications in biotechnology and bioremediation, in which phosphate monoesters are nonspecifically hydrolysed under alkaline conditions to yield inorganic phosphate. The hydrolysis occurs through an enzyme intermediate in which the catalytic residue is phosphorylated. The reaction, which also requires a third metal ion, is proposed to proceed through a mechanism of in-line displacement involving a trigonal bipyramidal transition state. Stabilizing the transition state by bidentate hydrogen bonding has been suggested to be the reason for conservation of an arginine residue in the active site. We report here the first crystal structure of alkaline phosphatase purified from the bacterium *Sphingomonas.* sp. Strain BSAR-1 (SPAP). The crystal structure reveals many differences from other APs: 1) the catalytic residue is a threonine instead of serine, 2) there is no third metal ion binding pocket, and 3) the arginine residue forming bidentate hydrogen bonding is deleted in SPAP. A lysine and an aspargine residue, recruited together for the first time into the active site, bind the substrate phosphoryl group in a manner not observed before in any other AP. These and other structural features suggest that SPAP represents a new class of APs. Because of its direct contact with the substrate phosphoryl group, the lysine residue is proposed to play a significant role in catalysis. The structure is consistent with a mechanism of in-line displacement via a trigonal bipyramidal transition state. The structure provides important insights into evolutionary relationships between members of AP superfamily.

## Introduction

Alkaline phosphatases (EC 3.1.3.1) hereafter referred to as APs, are widely distributed enzymes that hydrolyze the phosphate monoesters under alkaline pH conditions. APs are metalloenzymes and most of them, like *E. coli* PhoA (ECAP), require two Zn^2+^ ions per enzyme molecule for activity [1], [2]. A third metal is also found to be essential for optimum activity and in ECAP this metal is Mg^2+^ ion. Crystal structures of ECAP show that this Mg^2+^ ion is also part of the active site containing the bi-metallo-zinc core [2], [3]–[7]. X-ray structures of APs extracted from different sources show that despite the limited sequence identity of 30–50%, all APs are dimeric in nature, and contain similar α/β core structures with conserved metal binding sites [8]–[12]. The mode of action of APs has been well studied and a two stage in-line displacement mechanism proceeding through a trigonal bipyramid is proposed [2], [4]. In the first stage a phosphoenzyme intermediate is formed through nucleophilic attack on the substrate by the catalytic residue. In the second stage, this intermediate is hydrolysed to yield the products. The detailed molecular mechanism [2], [13] implicates one zinc ion in the activation and stabilization of the serine nucleophile, and the second zinc ion in the activation of a water molecule for attack on the phosphoenzyme intermediate [2], [13]. However, the role of the Mg^2+^ ion in the M3 site was subject of some discussion. One of the conserved features in all AP structures is the presence of an active site arginine residue, mutation of which results in a large decrease in the activity of APs. It has been argued that stabilization of the trigonal bipyramidal transition state through the pair of coplanar hydrogen bonds optimally made between the arginine guanidinium group and two nonbridging oxygen atoms of the transferred phosphoryl group contribute toward catalysis [14].

APs are part of a superfamily which includes nucleotide phosphodiesterases (NPP), co-factor independent phosphoglycerate mutases(iPGM), phosphonate monoester hydrolases (PMH) and aryl sulfatases(AS), among others [15], [16]. All these are metalloenzymes, but the number and nature of metal ions required is widely different within the AP superfamily [2], [3], [13], [16], [17]. NPP requires two zinc ions like ECAP, but has no requirement for the third metal ion. The bimetallic core in NPP is structurally identical to that in ECAP. Despite this striking similarity NPPs hydrolyze phosphate diesters with a preference of about 10^15^ fold over phosphate monoesters [16].

Recently we reported extraction of an AP, termed SPAP, from the bacterium *Sphingomonas* sp. strain BSAR-1 [18], exhibiting several unique features, such as constitutive expression, thermo-lability, extracellular release and high specific activity [19]. SPAP acts efficiently on a variety of substrates and can be inactivated by incubation at 60°C for 15 minutes. The specific activity of SPAP is significantly higher than the specific activity of most bacterial APs, such as ECAP. SPAP requires Ca^2+^ and Zn^2+^ for activity, while magnesium has no effect. SPAP over-expressed in a recombinant *E. coli* strain, was found to be very efficient in bioprecipitation of uranium as uranyl phosphate, under alkaline conditions [18]. Multiple sequence alignment shows highest similarity of SPAP with NPP instead of with APs. This comparison further predicts the catalytic residue in SPAP to be a threonine instead of the serine found in all other APs. Surprisingly, SPAP displayed very low phosphodiesterase activity. These observations are significant in tracing evolutionary relationships between APs and NPPs, and three dimensional structure of SPAP is expected to provide further insight. We have earlier reported successful crystallization of SPAP [19], and here we report the refined high resolution structure of SPAP bound by a phosphomonoester. In this crystal structure, while the bimetallo zinc system in the active site is similar to that of other APs, the catalytic residue is a threonine. The catalytically crucial arginine residue conserved in the active sites of other APs is deleted in the present structure. The structure thus suggests that SPAP represents a new class of APs, but with a molecular mechanism similar to that of ECAP. The structure also suggests that APs have evolved from a common ancestor molecule along two independent paths, one leading to ECAP and the other leading to SPAP. Structural similarities indicate closer evolutionary relationship between NPP and SPAP than between NPP and ECAP.

## Results and Discussion

Data collection and phasing statistics for SM-SPAP are given in Table 1. The crystals of SM-SPAP and native SPAP are tetragonal, contain one monomer per asymmetric unit, and belong to the space group P4_1_2_1_2 (Table 1 and 2). The refinement statistics for native SPAP are summarized in Table 2. The final contents of the asymmetric unit are: 526 protein residues (31–556), two zinc ions of partial occupancy (0.75), 278 water molecules, 5 glycerol molecules, 1 Ca^2+^ ion and one organic phosphate in the active site. The protein model has good stereochemistry with 98% residues lying in the favoured region of Ramachandran map.

**Table 1:** Data collections and phasing statistics for SM-SPAP.

Diffraction data	peak	edge		remote
_ _Unit Cell (Å)	a = 87.20	b = 87.20	c = 165.2	
_ _Wavelength (Å)	0.980159	0.980337		0.976261
_ _Resolution (Å)	1.85 (1.9–1.85)	1.85 (1.9–1.85)		1.9 (2.0–1.9)
_ _Space group	P4_1_2_1_2	P4_1_2_1_2	P4_1_2_1_2	
_ _No. of observed reflections	771391 (56396)	771426 (56386)		713097 (92791)
_ _No. of unique reflections	103688 (7964)	103898 (7994)		95461 (13050)
_ _Completeness	100 (100)	100 (100)		99.3 (95.4)
_ _Multiplicity	7.44 (7.08)	7.42 (7.05)		7.47 (7.11)
_ _R-sym^1^ (%)	7.3 (49.8)	7.0 (53.8)		8.0 (56.7)
_ _I/σ(I)	20.2 (4.13)	20.9 (3.82)		20.3 (3.89)

Phasing				
_ _refined f' (e-)	−7.1	−9.5		−2.1
_ _refined f'' (e-)	10.6	6.6		6.2
_ _mean figure of merit		0.58		
_ _number of Se sites		12		

The numbers between parentheses indicate the value in the outer resolution shell.

**Table 2:** Refinement statistics for native SPAP.

Structure refinement	
Unit Cell (Å)	a = b = 87.37, c = 168.16
Wavelength (Å)	0.980159
Resolution (Å)	1.95 (2.05-1.95)
Space group	P4_1_2_1_2
Total No. of reflections measured	379964 (44099)
No. of unique reflections	87058 (11547)
No. of free reflections	4420
Completeness	96.5%
I/Iσ	13.02 (2.15)
R_merge_ F	11.5% (71.0%)
R	15.53
R_free_	18.63
No. of protein atoms	4022
No. of solvent atoms	278
R.m.s deviation of bond lengths (Å)	0.007
R.m.s deviation of bond angles (°)	1.019
R.m.s deviation of dihedral angles (°)	16.27
Wilson B factor (Å^2^)	31.4

*The numbers between parentheses indicate the value in the outer resolution shell. R.m.s =  root mean square.

### Molecular Conformation

The first 19 of the 559 residues of SPAP are predicted to form the signal peptide, which is likely to be cleaved-off during protein secretion. Therefore the first amino acid of the full length secreted protein is numbered 20. In the present structure, visible electron density starts from the 31^st^ residue, suggesting that the first 11 residues from N-terminus of the secreted protein are very flexible. The overall shape of the protein can be described as that of a shallow cup with a thick base. The base and most of the wall region is made-up by one large domain, while the remaining portion of the wall is formed by a smaller domain. The larger domain, which comprises of residues mostly from the N-terminal region, consists of an eight-stranded mixed central beta sheet (β14, β2, β13, β10, β1, β9, β5, β6) surrounded by α-helices on the flat faces of the sheet. Of the eight strands, seven are parallel strands separated by one antiparallel strand (β13) in the middle, thereby giving this sheet N-terminal and C-terminal edges. The N-terminal edge of this sheet containing only loops is toward the base of the cup, while the C-terminal edge is toward the cup-opening. The smaller C-terminal domain primarily consists of a single two-stranded antiparallel beta hairpin covered by two α-helices on the surface. The relative orientations of the two domains is stabilised through hydrogen bonding interactions between loops from two regions of the two domains. The active site, located at the bottom of the wide and shallow opening of the cup is exposed to the environment, and this explains the highly non-specific nature of this enzyme.

**Figure 1:**
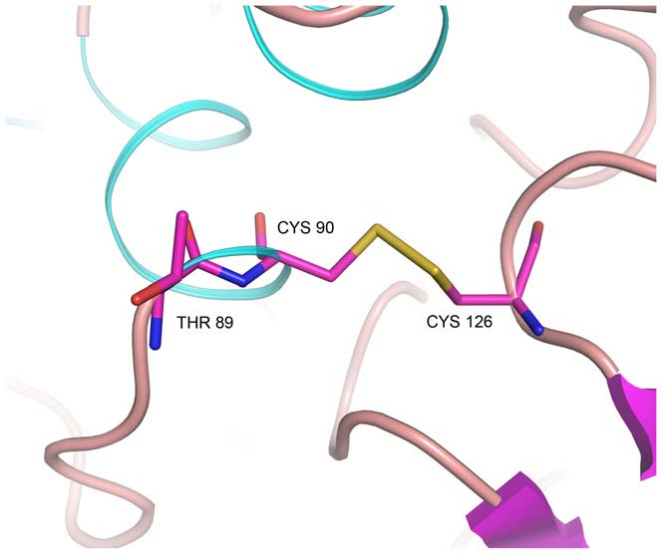
Unique disulfide bond near the active site: unique disulfide bond Cys90 – Cys126 next to the catalytic residue Thr89 may be crucial for the integrity of active site geometry.

There are six cysteine residues in the amino acid sequence of SPAP, and all of them are engaged in standard disulfide bonds in the present structure. Four of these cysteines (126, 231, 545 and 556) are in inserted regions of the amino acid sequence, when compared to ECAP. These disulfide bonds, Cys90- Cys126, Cys231- Cys314 and Cys545- Cys556, are crucial for the structure and activity of SPAP, since treatment with beta mercaptoethanol inactivates the enzyme with loss of secondary structure as analyzed by circular dichroism spectroscopy. The disulfide bond Cys90-Cys126, which is in the vicinity of the active site, might play a crucial role in maintaining the active site geometry by stabilizing the alpha helix containing the catalytic residue Thr89 (Figure 1). Both the cysteines are unique in SPAP. Cys90 is present next to the catalytic residue whereas in most other APs the residue next to the catalytic residue is an alanine except in PLAP where it is a glycine. Cys126 is part of an insertion which is not present in other APs. Cys231-Cys314 disulfide bond connects a big apha helix with a small 2 turn alpha helix present on the surface thereby stabilizing these alpha helices. These cysteines also have no counterpart in other APs. Third disulfide bond between cysteines 545 and 556 is located at the C-terminal end of the protein. This bond allows C-terminal end to form a cup like shape which interacts with the flap like region of other monomer through a Ca^2+^ ion to form the crystallographic dimer.

### Crystallographic Dimer

**Figure 2:**
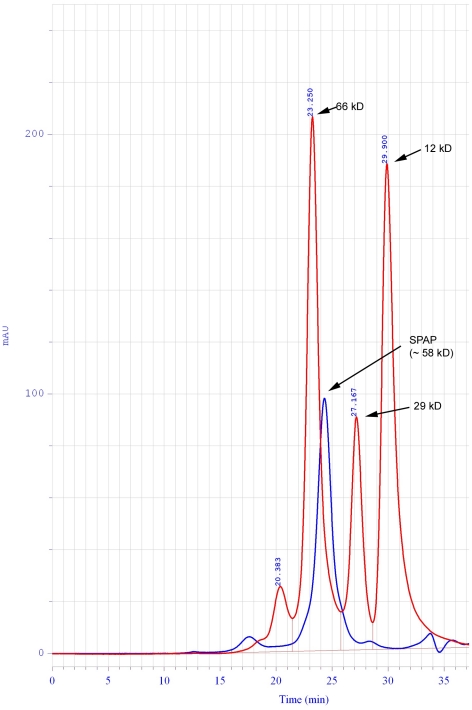
Size Exclusion Chromatography Elution profile of SPAP. Standards used are shown in red along with their molecular weights. SPAP, shown in blue, elutes as a monomer of molecular weight 58 kD.

SPAP crystallizes with one molecule in the crystallographic asymmetric unit, and crystal contacts lead to visualisation of a dimer. The total surface area buried upon dimerisation is 4030 Å^2^, which is 9.9% of the total accessible surface area (36570 Å^2^). It may be pointed out that a larger portion of the surface area is buried on dimerisation in other AP's: (TAP – 14%, ECAP, PLAP and SAP – 22%). Interestingly, SPAP elutes as an active monomer in size exclusion chromatography experiments (Figure 2), indicating that the dimerisation seen in the crystals is a crystallization artifact.

### Active Site and Catalytic Residue

**Figure 3:**
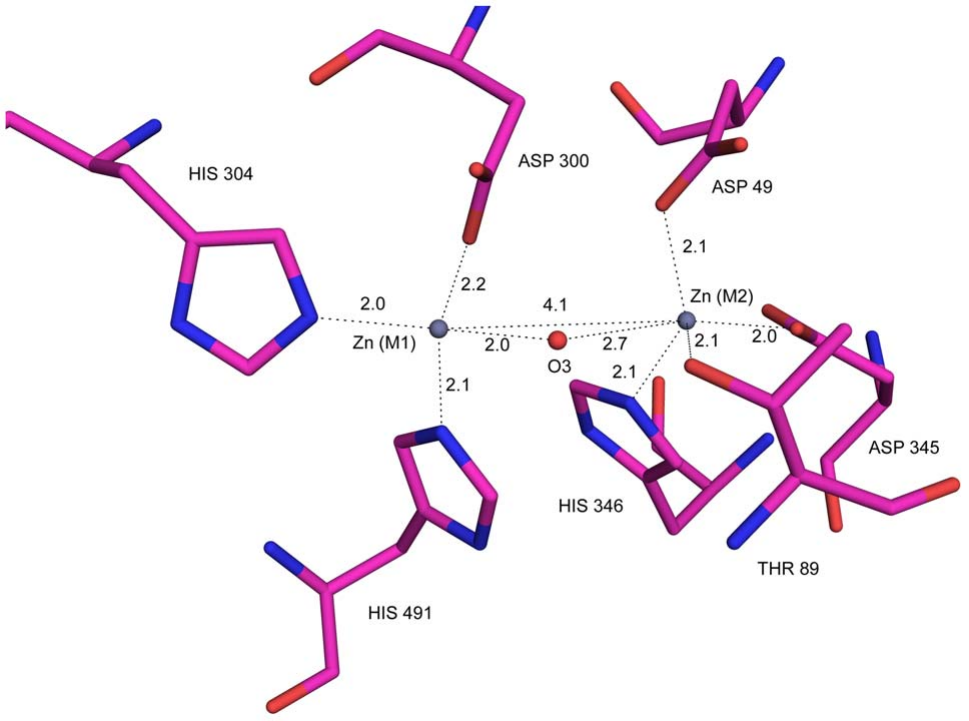
Active site geometry: co-ordination of zinc metal ions (slate) to protein residues (magenta) and to the substrate in the active site. The oxygen atom (O3) from the substrate-phosphate is shown in red.

The active site and the catalytic residue of SPAP were identified through structural superposition. Interestingly, despite poor sequence similarity, geometry of the active site of SPAP is identical to that in ECAP. The two active-site metal ions (M1 and M2), which were identified as Zn^2+^ through X-ray fluorescence measurements, are 4.1 Å apart. Zn^2+^ ion at M1 site is in coordination to polar atoms from His 491, His 304 and Asp 300, while Zn^2+^ at M2 site is coordinated by His346, Thr89, Asp345 and Asp49. The zinc coordination residues and distances are given in Table 3 and Figure 3. Structural superposition confirmed the catalytic residue in SPAP as Thr89, which was also suggested by sequence alignment. Thr89 superposes very well with Ser102 of ECAP with rmsd between Cα atoms being 0.69 Å. SPAP is a unique AP to have threonine as a catalytic residue, because in all other APs, Serine is conserved as a catalytic residue. Mutation of Serine to Threonine is not tolerated in ECAP [7].

**Table 3:** Metal coordination distances in SPAP and ECAP.

Metal	SPAP	ECAP(1ALK)
Zn M1	D300 OD1 (2.2)	D327 OD1(2.3)
	D300 OD2 (2.6)	D327 OD2(2.5)
	H304 NE2 (2.0)	H331 NE2(2.1)
	H491 NE2 (2.1)	H412 NE2 (2.0)
	Sub O3P (2.0)	
Zn M2	D49 OD1 (2.1)	D51 OD1 (2.0)
	T89 OG1 (2.1)	S102 OG (2.2)
	D345 OD2 (2.0)	D369 OD2 (1.8)
	H346 NE2 (2.1)	H370 NE2 (2.0)
	Sub O3P (2.7)	

### Michaelis Complex

**Figure 4:**
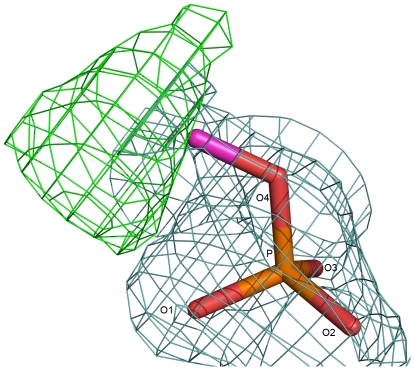
Michaeli's complex: electron density for the R-O-PO3 monoester substrate. Green contours are for the mFo – DFc map contoured at 3.0 σ level. Blue contrours are for the 2mFo – DFc map contoured at 0.8 σ level.

**Figure 5:**
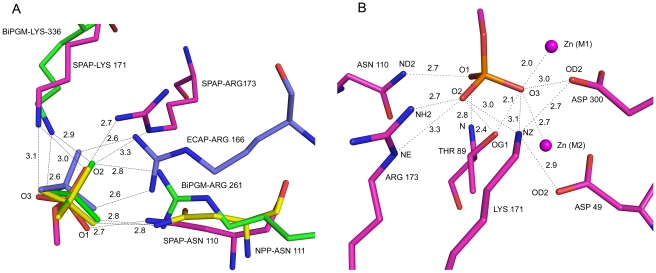
Interactions to the Phosphate moiety in the active site: (A) Structural superposition of residues binding the substrate in the AP superfamily: SPAP (Magenta), ECAP (slate), NPP (yellow), iPGM (green). Only SPAP has residues covering other members of the superfamily. (B) Phosphate interactions in the active site of SPAP. The distances given are in Å unit.

**Figure 6:**
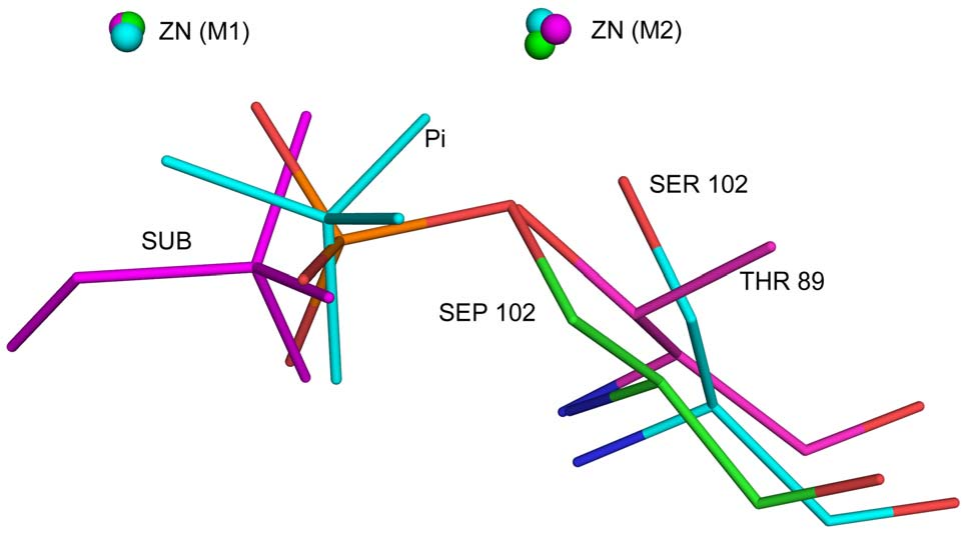
Relative orientation of hydroxyl group of catalytic residues at different stages: structural superposition in present structure (magenta), phosphoenzyme intermediate (green) and product complex (light blue). Note that Thr89 hydroxyl overlaps the ester oxygen in the phosphoenzyme complex.

Unaccounted electron density was observed in the active site region, and the volume of this density is larger than that for an inorganic phosphate, and therefore, we have interpreted this density to represent a phosphomonoester, ROPO_3_, substrate (Figure 4). The R-group could not be identified based on the shape of electron density, perhaps indicating its high flexibility. This monoester must have been picked up during protein production in *E.coli* cells. We have built the terminal C-O-PO_3_ portion of the substrate in the electron density map. The positions of the phosphate oxygens were confirmed through simulated annealed omit maps calculated by omitting one oxygen atom at a time. One of the nonbridging phosphate oxygens, O3, is sequestered between the two zinc ions, while the remaining two, O1 and O2, interact with residues Thr89, Arg173, Asn110 and Lys171 in the active site (Figure 5A and B). The distances of the two zinc ions from O3 are unequal, distance to the Zn1 ion being shorter at 2.0 Å. As already mentioned, hydrolysis by APs proceeds in two stages: formation of a phosphoenzyme intermediate and hydrolysis of this intermediate into products. The two stages of the reaction are expected to have a transition state of similar geometry. Three dimensional structures relevant to the second stage of the reaction show that there is a change in the conformation of catalytic serine residue as the reaction progresses [2], [4], [20], [21]. The torsion angle around C_α_ – C_β_ bond changes from −55° in the phosphoenzyme (Figure 6) to −77° in the product complex. As a result, the serine hydroxyl is pointing away from the phosphate in the product complex, while it is pointing toward the phosphate in both the phosphoenzyme and the transition-state complex. In SPAP, the corresponding torsion angle is −60°, and the hydroxyl of Thr89 is pointing toward the phosphate (Figure 6), thus confirming that the present structure is not a product complex, but is a Michaelis complex relevant to the first stage in which the phosphoenzyme is formed. In this complex, the nucleophilic hydroxyl, Thr89(OH), is at a distance of 2.6 Å from the target P atom, and the angle Thr89(OH)…P…OR is 170^0^, showing feasibility of in-line displacement mechanism through a bipyramidal transition state. A molecular model for the SPAP/transition-state complex was built by perturbing the present structure subject to the condition that the metaphosphate equatorial plane be perpendicular to the line joining Thr89 hydroxyl and the substrate ester oxygen. It was found that, in going from the present Michaelis complex structure to the transition state, the position of the phosphorous atom has shifted by about 0.8 Å, whereas the equatorial oxygen atoms have had to move much less (0.2 Å). The hydrogen bond between amide nitrogen of the catalytic residue and non-bridging phosphoryl oxygen becomes shorter in the transition state model, implying a possible role for this hydrogen bond in catalysis. Interestingly, some of the experimental kinetic data on hydrolysis of phosphate mono- and diester substrates, it is pointed out, can be rationalized by invoking a similar hydrogen bond between the catalytic amide group and the nonbridging phosphate oxygen atom [22].

### Lys171 – A Substitute for the Third Metal?

**Figure 7:**
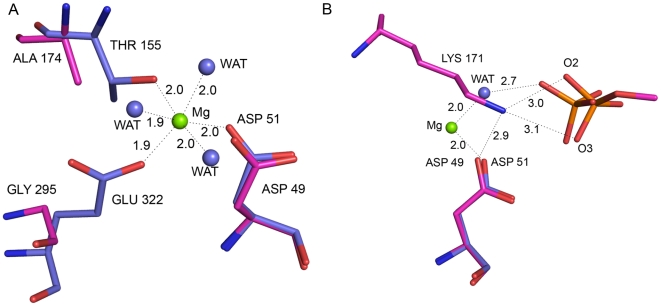
Lys171 in SPAP, a substitute for third metal: Superposition of SPAP (magenta) and ECAP (cyan). (A) amino acid substitutions that abolish Mg^2+^ binding site in SPAP. (B) Comparision of interactions of Mg^2+^ (ECAP) and Lys171 (SPAP) with substrate phosphate and active site aspartate.

In all ECAP-like AP structures, a third metal binding site is conserved. In ECAP this site is occupied by Mg^2+^ ion. The Mg^2+^ ion is coordinated octahedrally by Asp51, Thr155, Glu322, and three water molecules. Based on biochemical and structural evidences [22]–[24], three possible catalytic roles have been suggested for the Mg^2+^ ion: (i) activate catalytic serine by extracting its hydroxyl proton through coordinated water molecule, (ii) increase the charge in the active site through coordination to the active site aspartate residue, and (iii) stabilize the transferred phosphoryl group through coordination to phosphate oxygen atoms. The residues Thr155 and Glu322 in ECAP are replaced by Ala174 and Gly295 respectively in SPAP (Figure 7A). These changes abolished the third metal coordination site, and therefore there is no third metal bound to SPAP inthe active site. Interestingly, a lysine residue is recruited into the active site of SPAP, and the position of this residue is such that the positively charged NZ atom almost overlaps the water molecule coordinating the Mg^2+^ ion in the ECAP structure (Figure 7B). The suggestion can be made that Lys171 in SPAP may be a substitute for the Mg^2+^ ion in ECAP. Interestingly, a very similar situation exists in iPGM, where a lysine interacts with one of the nonbridging oxygen of the phosphate instead of Mg^2+^ bound water molecule. Substitution of lysine residues for metals has been observed also in AS [25]. If this hypothesis of substitution is correct, then interactions common to Lys171 NZ atom and Mg^2+^ ion are functionally important, and would throw light on the function of the third metal ion in ECAP-like APs. In the structure of SPAP, there is no direct or indirect interaction of Lys171 NZ atom with the catalytic residue Thr89, thereby suggesting that Mg^2+^ is unlikely to play the role of a general base, listed as the first option above. The interaction between Lys171 NZ and Asp49 OD2 atom in the active site of SPAP is similar to that of Mg^2+^ ion with Asp51 in ECAP. Further, Lys171 NZ atom in SPAP hydrogen bonds to nonbridging phosphate oxygen atoms, also like the magnesium-bound water molecule in ECAP. These observations suggest that Lys171 in SPAP and Mg^2+^ ion in ECAP, might be acting by increasing the positive charge in the active site (option 2 above) and/or by stabilizing the transferred phosphoryl group (option 3 above).

### SPAP Compared to Other APs

**Figure 8:**
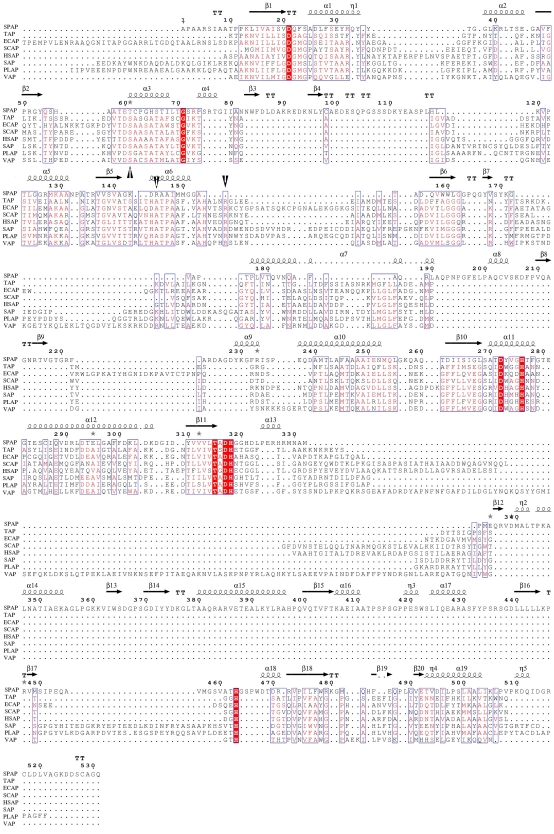
Sequence alignment derived from structural superposition of alkaline phosphatases. Absolutely conserved residues are shown in red. Three interesting facts are highlighted by black arrows: 1) presence of Thr89 in place of catalytic serine, 2) presence of novel Arg173 and 3) deletion, in SPAP, of conserved arginine.

The three dimensional structure of SPAP was aligned on known structures of other APs, using the SSM server [26], and the results are given in Table 4 and Figure 8. The average RMSD value for superposition of SPAP on the AP structures is 3.14 Å, which is much higher than the average RMSD values of 1.3 Å obtained when other APs are superposed among themselves. Out of 37 secondary structure elements only 12 could be superposed to yield reasonable RMSD's mentioned above showing that tertiary structure of SPAP is quite different. Superposing only the bimetallic zinc core and zinc binding residues yielded an RMSD value of about 0.4 Å (Table 4), showing that the bi-metallocore structure is conserved. The structure-based-sequence-alignment (Figure 8) indicates that major insertions, when compared to ECAP, are SPAP-residues 103-146, 220-244, 363-468 and 535-559, which add up to about 35% of the protein sequence. The alignment also points to absolute conservation of eight residues (D49, G99, D300, H304, T343, D345, H346 and H491). Most of these residues are, expectedly, in the active site, like D49, D300, H304, D345, H346 and H491. There are few residues which are conserved among all other APs compared but are different in SPAP. One such residue is Thr89, which aligns with the catalytic residue Ser102 in ECAP. The second residue is the characteristic active site arginine (Arg166 in ECAP), which is deleted in SPAP. This is a very significant difference because this arginine is postulated to stabilize the transition-state through bidentate hydrogen bonding. Though SPAP contains an arginine residue (Arg173) in the active site, the relative position and orientation of this arginine residue are very different (Figure 5A). Infact the amino acid residue in all other APs at this position is an alanine (ECAP Ala 154). The separation between Cα (Arg173) and Cα (Arg166 ECAP) is 6.64 Å, when ECAP and SPAP structures are superposed. Arg173 is unable to make the bidentate pair of coplanar hydrogen bonds with two non-bridging oxygens of the phosphoryl moiety that is observed in the ECAP structure. Instead, it forms a single hydrogen bond with one nonbridging oxygen atom, O2. A hydrogen bond to the second nonbridging oxygen atom O1 is formed by Asn110. These two hydrogen bonds are not coplanar. Therefore, the interaction pattern observed in SPAP could be a different way of stabilizing the bi-pyramidal transition state selected by nature during evolution.

**Table 4:** Structural comparison of SPAP with other APs; only C_α_'s are used to superpose.

Superposed onto structure(PDBID)	RMSD		% identity
	Whole subunit	active-site	
TAP (2IUC)	3.17	0.459	14.2
ECAP(1ALK)	3.24	0.392	14.6
SCAP(3A52)	3.10	0.366	11.6
HSAP(2X98)	3.11	0.529	14.2
SAP(1SHQ)	3.14	0.440	13.7
PLAP(1ZEF)	3.17	0.482	12.4
VLAP(3E2D)	3.07	0.447	12.0

Number of SS elements aligned  =  12.

Maximum number of residues aligned  =  233.

**Figure 9:**
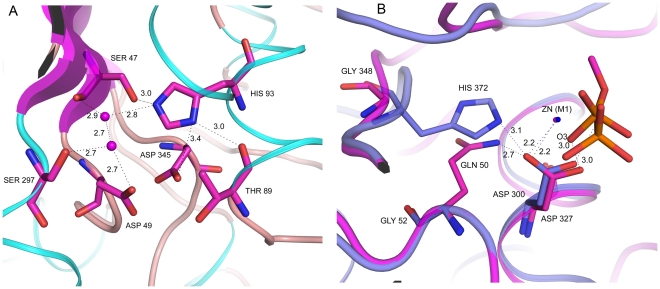
Novel hydrogen bonding interactions in SPAP: (A) Hydrogen bonding network involving His93 (B) Conservation of hydrogen bond from Asp300 OD1 through correlated sequence differences in SPAP (pink) and ECAP (slate). Distances are in Å unit.

The structure based sequence alignment also shows that there are few residues which are present in SPAP but not seen in any ECAP like APs. Interestingly, these residues are present in different members of AP superfamily. For example, the residue Asn at position 110 has no counterpart in ECAP and other APs, but is part of the active site in NPP and PMH. Similarly, the residue Lys171, though not present in NPP, ECAP or any other APs, occurs in phospho-gluco-mutase from *Bacillus stearothermophilus* (BiPGM). Another one is His93, which replaces alanine in this position in other APs, but is present in NPP. His93 stabilizes the position of active site Thr89 by forming bridging hydrogen bonds with the carbonyl oxygen of Thr89 and hydroxyl of Ser47 (Figure 9A). His93 also binds to the zinc-binding residue Asp49 indirectly through water-mediated hydrogen bonds. Functional importance of these interactions requires further investigation.

### Substrate Specificity

As already mentioned, sequence and structural comparisons show that SPAP is more similar to NPP rather than to other APs, and yet, it displays substrate preferences characteristic of APs. Therefore, the structural differences in the active sites of NPP and SPAP together with structural similarities in the active sites of ECAP and SPAP could provide insights into the 10^15^ fold difference in specificity between APs and NPP. Since the bimetalloactive site in SPAP is structurally identical to that in ECAP and in NPP, the specificity difference must be due to some other factors. Similarly, the residues Asn110 in SPAP and Asn111 in NPP are equivalent, and form a similar hydrogen bond with nonbridging phosphate oxygen atom, thus ruling out Asn111 as contributing toward specificity of NPP. Through kinetic and site directed mutagenesis studies [16], [22], the following factors in substrate binding have been estimated to account for a specificity difference of about 10^12^: i) bidentate hydrogen bonds to phosphate oxygen atoms from catalytic arginine in APs, ii) indirect third metal binding through water molecules in APs, and iii) binding to the diester R' group in NPP. In SPAP there is no catalytic arginine residue forming bidentate hydrogen bonds with the phosphate monoester. However, two hydrogen bonds to the phosphate oxygens are made by two separate protein residues, Arg173 and Asn110. Though there is no Mg^2+^ ion near the active site of SPAP, there is a positively charged lysine residue, Lys171, in the active site which binds directly with the nonbridging phosphate oxygen atom from the substrate (Figure 7B). NPP has a binding site for the R' group of phophodiesters, which is blocked in APs by active site Arginine and other amino acids. Even though SPAP closely resembles structurally NPP, in SPAP also this site is sterically blocked by Lys171, Arg173 and Tyr301. Thus, in these three respects interactions in the active site of SPAP resemble broadly those in ECAP but differ from those found in NPP, and therefore the three factors listed above would be appropriate factors to explain the specificity difference. A specificity difference of about 10^3^ fold is still unaccounted for. The present structure reveals another hydrogen bond near the active site, which also could contribute toward substrate specificity. This hydrogen bond is conserved in other APs, but is not observed in NPP. This is the hydrogen bond in which side chain of Gln50 is a donor and Asp300 OD1 is an acceptor (Figure 9B). Interestingly, this hydrogen bond is conserved despite deleterious substitution of the donor residue. In ECAP, the donor atom is from the side-chain of His372 (ECAP) residue. In some other ECAP-like APs the donor residue is a threonine. But in SPAP, the residue equivalent to His372 (ECAP) is a Gly348, which has no side chain donor atom. The deleterious effect of this difference is annulled by the compensatory difference at a different position in the sequence: Gly52 (ECAP)/Gln50. There is no change in the conformation of Asp300, but the side chain of Gln50 reaches out and hydrogen bonds with Asp300 OD1. Interestingly, this hydrogen bond is observed also in BiPGM [27] and phosphopentomutase (PDB id 3M7V), both members of the AP superfamily, where the substrate is a monoester like in AP. Asp300 OD1 also binds Zn1 ion (Figure 5B and 9B). The combined effect of these two interactions from Asp300 OD1 would be to draw electronic charge away from Asp300 OD2 atom, thereby reducing the repulsion between Asp300 OD2 and the phosphate oxygen O3 (Figure 5B). In NPP, on the other hand, where this repulsion would be less because of the lesser negative charge in the phosphoryl group, the corresponding hydrogen bond from an equivalent Asp residue is absent [16].

### SPAP Represents a New Class of AP

There are several features which make SPAP a novel AP. First, the key catalytic residue in SPAP is Thr89 instead of serine found in all other APs. Second, the conserved third metal ion binding pocket in the active site in ECAP-like APs, is abolished in SPAP (Figure 7A and B). Third, the key active site arginine (Arg166 in ECAP) is deleted in SPAP. Fourth, the sequence identity between SPAP and ECAP-like APs ranges from 12% to 14.6% (Table 4), which is very low compared to the range of 30% - 50% observed among ECAP-like APs. Fifth, the combined presence in the active site of key amino acid residues Thr89, Asn110, Lys171 and Arg173, observed only separately in the active sites of members of the AP superfamily. Interestingly, the relative positions and interactions of these residues are maintained in the individual active sites (Figure 5A). For example, the Cα atom of Asn110 overlaps the Cα atom of Asn111 (NPP) to within 1.2 Å, while the Cα and NZ atoms of the two lysines (Lys171 and Lys336 (BiPGM)) superpose to within 1.3 Å and 0.69 Å respectively. Similarly, the Cα's of Thr89 and its counterpart in NPP superpose to within a distance of 0.4 Å. In NPP, Asn111 forms a hydrogen bond with a nonbridging phosphoryl oxygen atom as observed in the present structure. Similarly, the lysine residue in BiPGM corresponding to Lys171, hydrogen bonds to two nonbridging oxygen atoms, again as observed here. All the features described above suggest that SPAP represents a new class of APs. The signature of this new class would be the combined presence in the active site of residues equivalent to Thr89, Asn110, Lys171 and Arg173. To explore for other members of this new class a BLAST search was done within the NR database, using SPAP sequence as a query. The result contained many hits which had been annotated either as NPP or as AP. Sequence alignments of these hits were examined for combined presence of residues equivalent to Thr89, Asn110, Lys171 and Arg173. Surprisingly, many of these proteins contained the above mentioned residues together, showing that there will be several members belonging to this class. It is also conceivable that many proteins presently annotated as NPP may actually turn out to be APs similar to SPAP.

### Implications toward Enzyme Evolution

It is an established fact that proteins in a superfamily evolve to acquire novel functions and structures through mutations at key positions. Sequence homology between different members of the superfamily is therefore often very low. In such conditions structural alignment method can provide better understanding of molecular evolution. SPAP is a member of AP superfamily. Structural superposition has suggested that SPAP might be evolutionarily more related to NPP than ECAP like APs. In order to further investigate evolutionary relationship of SPAP in the AP superfamily, we used the DALI server [28]. DALI server analysis has given some interesting results. It confirms that SPAP has highest similarity with NPP (Z-score 28.2) rather than with ECAP (Z-score 20.1) or other APs. Surprisingly, the scores for comparison with PMH, AS and iPGM are also higher than that for APs (25.2, 23.4 & 21.9 respectively). These observations and the unique combination of active site residues suggest that different members of the AP superfamily evolved from a SPAP-like ancestor, by retaining in the active site only the amino acid most relevant to a given activity, while mutating out others in the process of specializing toward that activity.

The differences in the pattern of enzyme-substrate interactions in the active sites of SPAP (present work) and ECAP suggest that nature has found at least two solutions to recognition and hydrolysis of phosphate monoesters under alkaline conditions, one as embodied in the structure of SPAP and the other as embodied in the structures of ECAP-like APs. These solutions have to be considered as evolutionarily independent because, in the enzymes SPAP and ECAP, the catalytic residue type, metal ion requirements, molecular conformation, etc. are very different. The homology in the amino acid sequences of SPAP and ECAP is also very low (14.6%). Further, the amino acid residues involved in the interactions with the substrate come from topologically different regions in the two enzymes. So we suggest that the SPAP-like and ECAP-like APs could have evolved along different paths from a single ancestral molecule.

**Figure 10:**
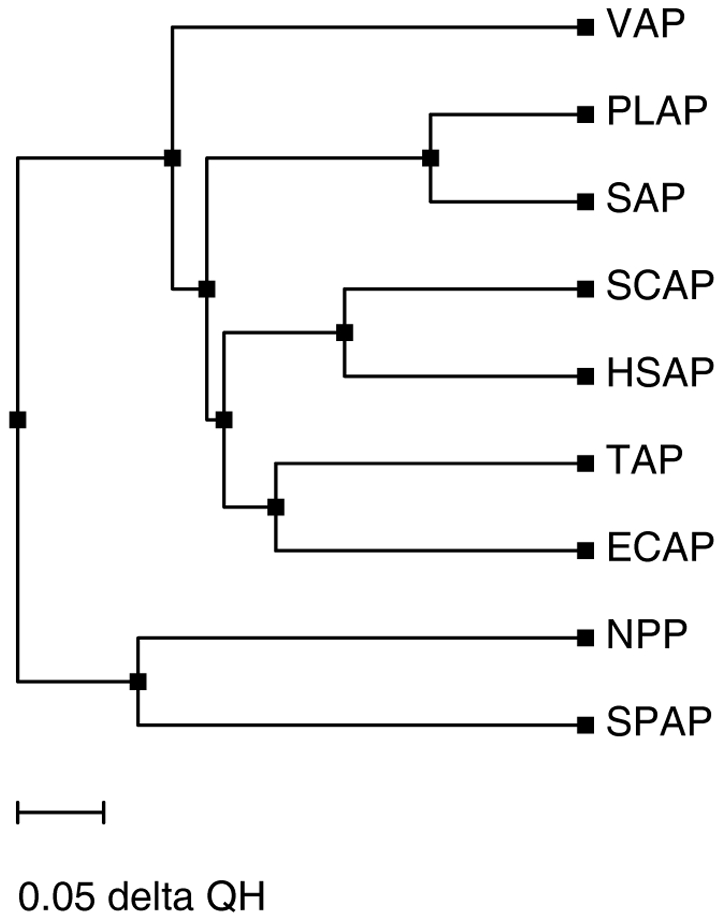
Structural superposition based cCladogram showing evolutionary relationship within AP superfamily (ECAP like APs, SPAP and NPP). Note that SPAP and ECAP-like AP's are on different branches of evolution tree. Also SPAP and NPP are predicted to have a common ancestor.

While analyzing evolutionary relationships between NPP and APs, Zalatan et al [16] have suggested the existence of an ancestral AP-like precursor, mutations into which introduced parts of an R' binding site that could have enhanced diesterase activity without decreasing monoesterase activity. We propose that the diesterase activity of NPP may be the result of evolution from the SPAP-like AP rather than from ECAP-like AP, for reasons given below: 1) the catalytic residue in SPAP and NPP is the same viz; threonine, 2) the RMSD for structural superposition of NPP onto SPAP is smaller (2.38 Å) than that onto ECAP (3.24 Å), 3) the number of residues aligned in structure comparison is higher for SPAP (320) as compared to ECAP (223), and 4) SPAP doesn't require Mg^2+^ ions for activity, like in NPP, whereas ECAP-like APs do require Mg^2+^ ions. Our hypothesis, therefore, is that ECAP and SPAP have evolved along different paths from a common ancestor, and that if NPP has indeed evolved from an AP-like ancestor, this ancestor is SPAP-like rather than ECAP-like. A phylogenetic tree of SPAP, NPP and ECAP based on structural superposition created using VMD [29] clearly support this hypothesis (Figure 10). In this tree SPAP and NPP form one group and all other APs form another group. This tree also gives some information about evolutionary trends among ECAP like APs but confirmation of that requires further study.

In conclusion, three dimensional structure of a substrate-bound alkaline phosphatase (SPAP) from the *Sphingomonas sp.* strain BSAR -1 bacterium has been determined to a resolution of 1.95 Å. The structure suggests that SPAP belongs to a new class of APs. The catalytic residue is a threonine rather than a serine, and the magnesium binding pocket observed in the active site of all other APs is abolished in SPAP. When compared to ECAP-like APs, the architecture of the active site is similar only to the extent that a bimetallic zinc core is coordinated by conserved His and Asp residues, while the combination of residues (Arg173, Asn110 and Lys171) binding the substrate phosphoryl group is different. Individual residues of this combination are observed only separately in the active sites of different members of the AP superfamily. The orientation of the phosphoryl moiety in the active site is consistent with the in-line displacement mechanism mediated by a trigonal-bipyramidal transition state. The characteristic pair of hydrogen bonds between active site arginine and nonbridging posphoryl oxygens from the substrate is replaced by two non-coplanar hydrogen bonds contributed by Asn110 and Arg173. This mode of interaction may be an alternate way of stabilizing the bi-pyramidal transition state in alkaline phosphatases. A positively charged lysine residue, Lys171 recruited for the first time into the active site of an AP interacts directly with two nonbridging phosphoryl oxygens, and this could lead to enhanced catalysis. The structure is suggestive of independent evolutionary paths for SPAP and ECAP-like APs, and also of the likelihood that NPPs evolved from SPAP-like structure rather than from ECAP-like structure. The structure can be used to identify residues that can be modified to engineer SPAP for molecular biology applications.

## Materials and Methods

### Overexpression and Purification of SPAP protein

SPAP protein was overexpressed and purified as mentioned earlier [18], [19]. Briefly the recombinant *E. coli* strain overexpressing SPAP was grown aerobically at 37°C in Luria-Bertani (LB) medium and the culture was induced by 1 mM IPTG at 30°C for 4 h. The cells were lysed by sonication (Branson, Germany) in 100 mM Tris – 100 mM NaCl buffer containing 0.5 M urea. The enzyme was purified using Ni^+2^-nitrilotriacetic acid affinity chromatography. A selenomethionine derivative of the protein(SM-SPAP) was also expressed and purified similarly except that the *E. coli* strain BL21 (DE3) carrying *SPAP* gene insert was grown in M9 medium supplemented with selenomethionine. The purified recombinant protein was dialyzed against 50 mM Tris −100 mM NaCl buffer in cold (4°C). The protein was then concentrated using centricon centrifugal concentrator (10 kD cut-off). The purity of the protein was determined after resolving it by SDS-PAGE on 10% resolving gel and by Coomassie Brilliant Blue (CBB) staining.

### Size exclusion chromatography of SPAP

The Superdex^TM^ 200 10/300 GL chromatography column from GE Healthcare and JASCO HPLC (Model PU2089) were used to carry out size exclusion chromatography of the purified protein. The elution buffer used was 20 mM Tris, pH 8.0 containing 150 mM NaCl. The flow rate was 0.5 ml per minute and cytochrome-c (12.4 kD), carbonic anhydrase (29.0 kD) and Bovine serum albumin (66 kD) were used as standards.

### Activity measurements

Catalytic activity of eluted SPAP fraction was checked, at room temperature, by using the spectroscopic method with pNPP as the substrate. The release of pNP after addition of 0.05 microgram protein to 3.0 ml of reaction buffer (pH 8.5) was monitored at 405 nm for a period of 20 minutes.

### Crystallization of SPAP

Crystallization was performed at 298 K by sitting-drop vapor diffusion method in 96-well crystallization plates (Greiner, 3cup) using Cy-Bio HTPC robot. Initial screening was performed by using, as reservoir solution, the commercially available crystallization screens (Molecular Dimensions Ltd, Emerald Biosystems and Jena Biosciences). Both native as well as SM-SPAP crystallized under several conditions. Crystallization condition for SM-SPAP was optimized manually by hanging-drop vapour diffusion method in 24-well crystallization plate. Best SM-SPAP crystals were obtained when the precipitant was 2M ammonium sulfate. Native crystals used in the present study were grown under following chemical conditions: (1.6 M ammonium sulfate, 10% dioxane, 0.1 M MES buffer at pH 6.5).

### X-ray Diffraction Data collection & Structure Refinement

X-ray diffraction data were collected at 100 K on both native and SM-SPAP crystals by using the oscillation method and the FIP beamline on the European Synchrotron Radiation Facility (ESRF) [30]. The crystals were dipped for 10 – 20 seconds in the cryoprotectant solution (mother liquor containing 25% glycerol) before flash freezing in liquid nitrogen. A fluorescence scan on SM-SPAP crystals enabled identification of the three wavelengths to use for a MAD data collection. At each wavelength, 180 diffraction images, each for an oscillation angle of 1° and an exposure time of 60 seconds, were recorded. For native crystals 180 diffraction images at a single wave length were recorded, each for an oscillation angle of 1° and an exposure time of 60 seconds.

### Structure Determination

The oscillation frames were processed using XDS [31]. Selenium sites identification, MAD phasing and initial model building were performed using the AutoSol tool in the software suite PHENIX [32]. Extension of the model obtained from AutoSol was carried out using the AutoBuild tool of PHENIX [32], [33]. The model was improved manually in the experimental electron density maps displayed using software packages Coot [34], [35]. Water molecules added using phenix-refine, were edited manually by examining the electron density at each site. The software phenix-refine was also used to execute standard protocols of simulated annealing (SA) refinement [32]. During the final stages, TLS refinement of the molecular model was carried out as implemented in PHENIX [36], [37]. The native structure was later solved by using the protein part of SM-SPAP structure as a search model in Molecular Replacement calculations using AutoMR tool of PHENIX [32]. The procedures used for refinement of the native structure were same as described above. All figures were prepared by using the software PyMol [38] except structural alignment figure, which was prepared by ESPript server [39] and cladogram which was prepared by VMD [29]. Atomic coordinates and structure factors have been deposited in the Protein Data Bank under the PDB Id 3Q3Q.

### Structure Analysis

The stereochemistry of the structure was analysed by ADIT deposition tool of PDB. DALI Lite server [28] was used to identify structurally similar proteins from PDB. Structural comparision was done using Secondary Structure Matching (SSM) server [26]. Among those identified, PISA server [40] and PIC server [41] were used to analyse dimer interface.

### Phylogenetic Analysis

A BLAST run was done using NCBI within NR database using SPAP as query to indentify proteins similar to SPAP. In this BLAST results proteins similar to SPAP were identified by looking at the combined presence of some key residues. The structural alignment for phylogenetic analysis was carried out using the multiple structural alignment program STAMP integrated in the molecular visualization program VMD, version 1.8.7 [29] with the parameters npass = 2, scanscore = 0 and scanslide = 2. Phylogenetic trees were calculated using software MultiSeq [42]) in VMD based on structural similarity measure Q_H_ [43] which takes into account the effects of the gaps on the aligned portion.
